# Potential of water sediments in construction materials: Current approaches and critical consideration of future challenges

**DOI:** 10.1016/j.heliyon.2024.e41121

**Published:** 2024-12-11

**Authors:** Jan Fořt, Ayodele Afolayan, Václav Kočí, Lenka Scheinherrová, Jiří Jan, Jakub Borovec, Robert Černý

**Affiliations:** a Department of Materials Engineering and Chemistry, Faculty of Civil Engineering, Czech Technical University in Prague, Thákurova 7, 166 29, Prague 6, Czech Republic; b Biology Centre of the Czech Academy of Sciences, Institute of Soil Biology and Biogeochemistry, Branišovská 1160/31, 370 05, České Budějovice, Czech Republic

**Keywords:** Dredged sediment, Valorization, Building material, Replacement, Reuse, Aggregate

## Abstract

Human activities result in sediment accumulation, so the reservoirs gradually lose their functionality, impacting their ability to manage large flood inflows, supply water, and generate hydroelectric power. Therefore, periodic removal of sediments from water reservoirs is essential to maintain functionality. Notwithstanding, the management of dredged sediments is a multifaceted process that involves careful consideration of environmental, regulatory, and economic factors to ensure their responsibility and sustainable handling. In this regard, the search for synergies represents an important development factor in the current industrial world, which can bring several benefits, especially in the construction industry. By reusing sediments, the environmental externalities typically associated with building materials production can be reduced by transforming sediments from waste material into valuable resources. The consolidated knowledge in this review emphasizes the advances in the upcycling of dredged sediments into building materials in various ways, including aggregate production, brick manufacturing, traditional binder replacement, and alkaline activation. The provided summary of benefits, disadvantages, challenges, and future potential of freshwater dredged sediments (FDS) use can stimulate the rationalization of material flows, reduce the dependence on primary raw materials in the construction industry, and at the same time contribute to maintaining the functionality of water reservoirs.

## 1 Introduction

The deposition of freshwater dredged sediments (FDS) in reservoirs not only leads to the loss of storage capacity but also triggers a range of sedimentation-related issues. As sediment accumulates, the reservoir gradually loses its functionality, impacting its ability to manage large flood inflows, supply water, and generate hydroelectric power. It may also cause infrastructure instability and have an impact on the environment [1,2]. Consequently, the management of FDS becomes crucial, emphasizing the importance of regular dredging. This practice not only helps to maintain the reservoir's functionality but also makes substantial quantities of FDS available for efficient reuse. Therefore, the safe disposal or utilization of FDS generated globally remains an open challenge and requires significant attention.

Large volumes of FDS are generated globally, posing challenges for disposal. According to Beddaa et al. [3], about 600 million m³ of sediments are generated worldwide annually. In Europe alone, around 300 million tons of sediments are generated, as reported by Snellings et al. [4]. For example, France plans to dredge approximately 33.5 million m³ of river sediment over the next decade, with 413,000 m³ already dredged from rivers, canals, and tributaries in the Paris Region and its surroundings between 2015 and 2017 [3]. In the United States, an annual dredging volume of 200 million m³ was reported, incurring costs of about $1.3 billion [5].

However, part of the challenge associated with processing such large volumes of sediments is also their inhomogeneity and differences in composition in individual areas. Sediment exhibits considerable variability influenced by watershed and human activities. The elemental makeup primarily relies on the rock component from which it originates, and the characteristics may vary according to the economic activities in the area. Nutrients from agricultural runoff, waste water discharge, urban storm water, and contaminants such as hydrocarbons, micro plastics, chemicals, and trace metals with varied hazardous properties from industrial effluents can attach to FDS particles. Notably, the key components in FDS encompass minerals, microorganisms, pollutants, and organic matter [[6], [7], [8], [9]]. For instance, FDS contains a variable amount of organic matter; organic matter (OM) content greater than 30 % is regarded as high. FDS with high OM is of low applicability in the construction industry due to its negative impacts on the engineering properties of the material [[10], [11], [12], [13], [14]]. Therefore, it is of particular importance to distinguish the high-organic content sediment with a significant portion of phosphorus and nitrogen. Also, granulometry analysis to identify the percentage of sand and fine FDS with clay and silt content.

FDS is supersaturated with high water content which may vary from 150 to 200 %. It contains free water, interstitial water that is bound by surface tension to the FDS particles, capillary water adhering to particle surfaces via adsorption and adhesion, and chemically bound water. In addition, sediment contains clay and silt which may have a high capacity for water retention. This amount of water increases the volume of solid sediment making handling difficult [[13], [14], [15], [16]]. Therefore, dewatering dredged sediment is essential to greatly reduce the volume, which will significantly decrease transportation and disposal costs and promote the reusability of FDS [17].

Thorough FDS characterization is crucial to determine the most suitable disposal management process for each case or reuse option. The disposal of FDS raises concerns about the accumulation of contaminants While landfilling disposal is sometimes adopted, identifying suitable landfills near dredging locations poses a challenge. Namely, leaching contaminants may impact groundwater, thus establishing confidence that disposal won't have adverse environmental effects is essential [[18], [19], [20]].

However, varying national guidelines create a lack of uniformity in FDS management. Given the substantial global production of FDS and the evolving legal requirements, traditional practices like direct disposal in landfills are no longer feasible [21,22]. Proper landfilling is nowadays not allowed in most EU regions. However, understanding the practice of sediment disposal is very complicated; this topic would deserve to be considered as a principal goal of a specific manuscript. In general, the general principles are given in the Water Framework Directive (WFD 2013/39/EU of August 24, 2013), which is valid for all EU member states. The management practice of dredged sediment is described in the European Waste directive (75/442/EEC), which defines the circumstances when the material is treated as waste. The consequent disposal strategies are based on the level of material contamination according to the European Waste Catalogue. In case of the high content of dangerous substances, the material must be classified as 170505 – Dredging spoils containing dangerous substances. A threshold for labeling the material as hazardous is given in Directive 2008/98/CE of November 19, 2008, Annex III.

Moreover, landfilling has been shown to have an environmental impact over 10 times greater than taking no action, leading to site contamination, remediation operations, and post-remediation challenges [23]. Therefore, valorization and utilization of beneficial aspects of FDS, thereby turning it into a valuable resource, presents a more effective solution than considering FDS as waste to be disposed of. In this regard, useful components can be separated from FDS to reduce the need for extracting virgin materials from natural resources, contributing to the conservation of minerals, and other valuable components [24,25].

This research paper aims at a summarization of the possible utilization of dredge sediments in the construction industry to reveal the synergy between water reservoir maintenance, the upcycling of the produced sediments, and the replacement of traditional building materials. In this regard, this study aims at the identification of major challenges and highlighting of the main benefits to stimulate a significant shift in accepting FDS as a valuable material source. The benefits associated with FDS utilization are predominantly based on environmental and economic pillars. For example, the abundance and accessibility of FDS can contribute to the reduction in construction costs, enhancing economic viability while upholding circular economy principles. Utilizing FDS supports local material sourcing, lowers transportation needs and associated carbon emissions, thereby making construction more adaptable to local conditions [26,27]. The reuse scenarios for FDS mark a significant shift towards circular economy principles, promoting sustainability and efficient management of resources. Repurposing FDS for construction materials and other applications not only offers economic benefits but also reduces the reliance on conventional raw materials [28].

## 2 Water sediment management practice

Repurposing sedimentary materials across various applications contributes to sustainable resource management, offering an effective solution for disposal. From construction to agriculture, FDS utilization has gained attention as an innovative and eco-friendly material, raw FDS with a low water content of approximately 30 % has been used for agriculture, replenishment, and remediation. Coarse sediments are recycled as aggregates and fine sediments are used in ecological earthen bricks production, depending on their plasticity.

Industries and researchers are delving into the intrinsic properties of FDS to address challenges associated with waste management, environmental conservation, and the responsible utilization of natural resources. The exploration of multiple applications aims to harness its potential contributions to diverse sectors. However, the options for sediment reuse depend on its specific characteristics and the requirements of various industries [17,[29], [30], [31]]**.**

Specific ways of utilizing FDS have been described in the literature. A suitable FDS has been repurposed for soil filling [20,30,32,33], agriculture [31,[34], [35], [36], [37], [38]], coastal nourishment [[39], [40], [41]], and construction purposes [26,[42], [43], [44], [45], [46]]. Identifying appropriate applications minimizes waste generation and cost saving, and promotes principles of circular economy. Although the utilization of FDS presents potential advantages, there are conceivable challenges concerning high water content, contaminants, regulatory adherence, public perception, and technological advancements that need to be considered. Therefore, it is important to ensure the sustainability and safe utilization of FDS by developing effective and cost-saving methods to reduce the high water content and to mitigate the contaminants in sediments, and ensure they meet safety standards [39].

Several factors come into play when determining the suitability of FDS for various applications. For instance, for soil amendments or fertilizers, apart from low water content and solvability of the FDS, nutrient composition is important to consider. FDS rich in essential nutrients, such as nitrogen, phosphorus, potassium, and micronutrients, can provide valuable benefits for agricultural purposes [44,47]. FDS with a high pH level may require adjustments to enhance their suitability for optimal plant growth. To ensure the applicability of sediments for agriculture, it is essential to assess the levels of heavy metals, ensuring they fall within acceptable limits. Moreover, the presence of organic contaminants, such as pesticides, herbicides, or industrial chemicals, can significantly influence the suitability for agricultural use [31,36,47]. Construction represents an important sector where it is possible to use a large volume of material and thus contribute to reducing the sector's dependence on primary raw materials. Materials with a high proportion of inorganic components (SiO_2_, Al_2_O_3_, CaO, etc.) can be used here, in particular, whose use for selected applications results from the characteristics of the sediment [22,48].

## 3 Sediment valorization in building materials

### 3.1 General considerations

Transition to the circular economy, reduction in raw material consumption, carbon dioxide restrictions, lowering the environmental burden, and waste production are of particular concerns in the building industry [23]. The huge scale of the building industry further amplifies the needs of the sector in all mentioned issues. As the disposal of dredged sediments is viewed as a substantial issue, meeting these tasks can be considered as potential synergy between the material flows and the replacement of traditional materials in the building industry by the sediments. Furthermore, processing and modification of sediments into high-value materials complies with upcycling principles and allows more significant environmental benefits delivered by binder replacement [49,50]. As reported, the partial cement replacement may reduce the carbon footprint by up to 40 % and even more savings can be achieved by the application of FDS as the precursor in alkali-activated materials (AAMs) [22,51,52]. Such pathways are, however, not explored sufficiently, and the majority of the research remains aimed at low-grade materials. The utilization of sediments as aggregates or unfired bricks is accompanied by benefits related to the availability of the materials or lowered costs. However, environmental issues and advantages are not so evident. In this sense, the valorization of sediments in the building industry should be of particular importance, meeting criteria on environmental benefits and mitigation of waste production.

The utilization of FDS in building materials provides additional benefits in the form of the stabilization of the increased content of heavy metals thanks to the chemical precipitation of low soluble species [53]. During the hydration process of cement-based materials, heavy metals are encapsulated and solidified. The leaching of heavy metal is inhibited in such a system, and thanks to the long-term durability, the environmental risk is significantly reduced. A similar phenomenon can be identified for AAMs [54,55].

In the construction industry, several factors influence the potential application of FDS ranging from high water contents, material characteristics, as well as regulatory considerations. High water contents remain a challenge in sediment utilization. It affects the compaction, stability, and drainage characteristic of the FDS material. Therefore, it is important to dewater FDS for suitable reuse. Also, the construction industry requires the use of materials such as an aggregate of different particle sizes such as fine, sand, and coarse for strength and stability. FDS needs to meet the required particle size for specific material design. Construction materials have exact specifications and standards, and the need to meet these requirements is important to ensure the durability and safety of the built structures [43,56]. Permits may be required to use sediments for construction while adhering to environmental and safety standards [48,[57], [58], [59]]. The general requirements are given in Regulation (EC) No 1272/2008 of the European Parliament and of the Council of December 16, 2008 on classification, labeling, and packaging of substances and mixtures. In addition, the standards for the determination of functional parameters of designed materials should be followed. The products considered for the utilization in concrete must fulfill the requirements given in EN 934 Admixtures for Concrete, Mortar, and EN 480 Admixtures for Concrete, Mortar, and Grout – Test Methods. For example, the utilization as a partial cement replacement has to take into account the content of substances that may negatively affect the material performance, such as water-soluble chlorides. In this sense, for the use of marine sediments it seems to be problematic to keep their content below 0.15 % for reinforced concrete and 0.06 % for prestressed concrete. In addition, the limits for the total alkali content (Na_2_O + K_2_O) should not exceed 0.6 % to avoid alkali-silica reaction (ASR) [60].

In the ever-evolving construction industry, FDS use is a versatile resource with significant potential across diverse construction applications [26,42,43]. The utilization of FDS in the field of construction is driven by its unique properties and the industry's growing commitment to environmental responsibility. FDS exhibits versatile properties that can be tailored for specific construction needs, whether in the creation of structural elements or as a component in concrete production. Its adaptability positions it as a resource instead of waste [43,45,46]. While the potential benefits of utilizing in construction are significant, challenges exist. In particular, the high water content needs to be reduced, so that the material can be further used for alternative applications (high water content may limit further processing), also taking into account the large volume of sediments that must be processed. Although different dewatering techniques have been adopted, factors such as performance efficiency, processing time, and energy required to handle large volumes remain as obstacles. Currently, the existing dewatering technologies can be categorized into mechanical, biological, and chemical conditioning methods, while in general the dewatering performance is affected by FDS composition, dewatering device, and operating conditions [16]. The comparison between the methods and their performance influenced by various factors is presented in Table 1. Nevertheless, the natural dewatering technique is a sustainable, eco-friendly, and economical solution that can be attained by combining drainage and evaporation systems [3,61,62]. Drainage occurs fast and reduces the water content while the leftover FDS gradually evaporates to dryness. The rate of evaporation depends on the relative humidity, temperature of the surroundings, the granulometry of the dewatered sediment. Previous studies on natural dewatering include the findings of Hussan et al. [14] in a laboratory experiment that revealed that 34 % of water was drained in the first 12 h of testing. Even more distinct values were obtained by Azaiez et al. [63], who reported 32 %, 28 %, and 16 % moisture loss in 30 h, 25 h, and 8 h respectively.

**Table 1:** Comparison of dewatering methods.

Dewatering Methods	Technology	Application process	Pros	Cons	Hybrid methods.	Reference
Chemical conditioning	Flocculation using polyacrylamide, chitosan, tannic acid, composite-flocculant-FeCl_3_-PAM.	Water soluble polymers conditioners bind fine FDS particles by flocculation via bridging or charge neutralization. An increase in particle size improves solid retention and easy water removal.	Faster and high dewatering efficiency.	Polyacrylamide can have a negative impact on the environment due to toxicity and non-biodegradability. Not economical for large volume of FDS.	Hybrid with coagulant, geotextile tube, pressure filtration. .	[64] [15]
	Advance oxidation process (AOP) using		Bond water converted to free water.			
	Fenton (Fe_2_+/H_2_O_2_)		Reduced cost of management.			
Mechanical	Filter press, cyclone, centrifuge, clarifier.	Filtration under pressure removes water, leading to filter cake formation, while centrifuge separates liquid from solid.	Remove free water fast.	High installation, operational and maintenance cost.	Mechanical + flocculation, electro-dewater.	[65] [66]
				Equipment immobility.		
				High moisture content.		
				Bound water remains unremoved.		
Geotextile tube	Woven/unwoven polymeric material into tube shape.	FDS slurry is pumped into the porous fabric tube where the particles are retained and the water is drained out.	Cheap and efficient with minimal turbidity.	The choice of polymeric material must be tailored for each specific FDS condition. Mostly fabricated onsite. Energy is needed for pumping.	Flocculation + Geotextile	[[67], [68], [69]]
			Easy to fabricate and use.			
			Good for large volume of FDS.			
			It's more economical compared with mechanical dewatering.			
Biological	Bioleaching with wetland plants e.g. helophytes, aspergillus	Bacterial cultures are acclimated to FDS samples. They oxidize heavy metals in the FDS to soluble metal ions.	Simple, low operation and energy cost.	Bioleaching is a slow process.	Bioleaching + surfactant, AOP	[70,71]
	niger fungi.		Remove FDS turbidity, smell, pathogens and trace metals.	Less applicable for bulk FDS dewatering.		
	Bioleaching with sulfur or iron oxidizing bacteria.		High evapotranspiration rate by wetland plants.	Soil acidification is possible when oxidizing bacteria is used.		
Electrokinetic dewatering	Electro-osmosis, carbon electrodes, DC power supply.	Electrodes are arranged in a way that the cathode is placed below the filter medium while the anode is on top of the slurry, the negatively charged particles will be repelled by the cathode, reducing clogging of the filter medium. At the same time gravity will lead to passage of liquid through the filter, to some degree assisted by the electro-osmotic effect.	Removes both free and bound water.	High energy requirement.	Electro-dewatering + mechanical.	[16,72,73]
			Removes pathogens.			

The particle size and chemical and mineralogical composition are crucial parameters for assessing recycling options. In terms of particle size distribution, the particle size of 65 μm can be viewed as a threshold for further reuse alternatives [74]. Particles with bigger diameters, especially those rich in crystalline SiO_2_, are often used as sand replacement in concrete production [3]. Excluding the damping and landfilling, the utilization of fine sediments is more challenging, as only very limited established practice is implemented. The research scale of fine sediments includes reuse, replacement of raw materials for brick production, utilization as cement replacement and a potential precursor for alkaline activation. In this regard, the requirements for mineralogical composition are more specific [75,76]. The reaction capability of the material needs to be secured by additional thermal treatment to increase the content of amorphous (glassy) phases. The content of Al_2_O_3_ and SiO_2_ in reactive form determines the pozzolanic activity of sediments which thus can react with Ca(OH)_2_ in the concrete to form durable C-A-S-H reaction products similarly to other pozzolan-active materials. Based on results provided in Table 2, the fine freshwater sediments are mostly composed by SiO_2_ (40–55 %), followed by Al_2_O_3_ (13–17 %), CaO (7–14 %), and Fe_2_O_3_ (5–7 %). The majority of studied sediments fall within these intervals with only minor variations. However, these values cannot be easily used for the calculation of silicate modulus typical for the assessment of the material reactivity in the concrete industry due to low content of amorphous (reactive) phase. In this regard, understanding the mineralogical phases as well as the quantified results are of major importance. As reported by various authors, the quartz is the most dominant phase accounting for 20 %–46 % [[77], [78], [79]]. Notwithstanding, the quartz acts as filler, and cannot be accepted as a reactive form of SiO_2_ required for the pozzolanic reaction. Therefore, calculations based on XRF results do not provide reliable results and XRD analysis with Rietveld refinement needs to be used for the quantification of the reactive content. The content of clay minerals (kaolinite, illite) or feldspars refers to the potential of the material, when exposed to elevated temperatures, to increase its reactivity in a similar way as for calcined clays, metakaolin, and other clayey materials used in the construction industry. As reported in Fořt et al. [79], the increased content of Al_2_O_3_ correlates with the improved mechanical properties of the designed AAMs (after the calcination process).

**Table 2:** The oxide composition of FDS utilized in building materials.

Source	Al_2_O_3_	SiO_2_	Fe_2_O_3_	CaO	MgO	Na_2_O
Ferone et al. [80]	15.6	47.51	6.74	10.22	2.41	0.25
Messina et al. [74]	16.9	50.93	6.43	7.93	1.98	0.72
Mostefa et al. [77]	15.5	42.66	6.38	13.39	2.86	0.51
Liao et al. [81]	22.7	61.38	8.61	0.7	2.03	1.33
Faure et al. [82]	16.1	50.13	5.97	7.75	1.62	0.12
Aouad et al. [83]	13.7	44.48	5.82	14.58	1.78	0.59
Faure et al. [78]	11.9	46.99	5.77	12.13	3.02	0.84
Habibi et al. [84]	16.52	55.51	3.29	5.77	0.63	0.59
Chiang et al. [85]	15.45	70.14	5.37	0.64	1.64	1.02
Fořt et al. [79]	21.62	59.84	8.06	1.48	2.4	0.91
Afolayan et al. [86]	28.52	58.59	6.31	0.37	0.75	0.22

Other challenges affecting the beneficial reuse of FDS include ensuring the material meets standards for strength, durability, and safety. Rigorous testing and quality control measures are essential to guarantee suitability for construction purposes [21,43,46,87].

**Fig. 1:**
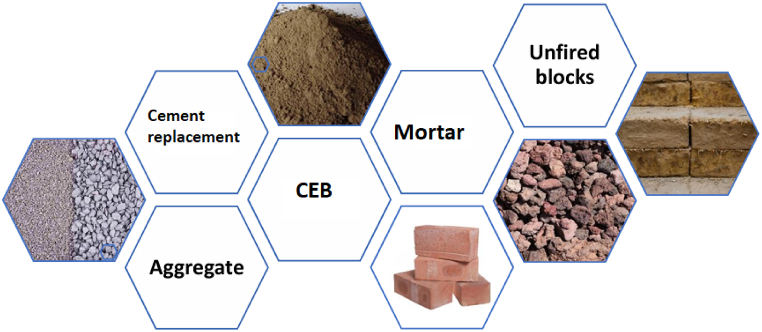
Options for sediment utilization in building materials.

Tailoring each application based on FDS attributes like water content, particle size, compaction characteristics, and mineral composition, allows for the customization of building materials. FDS incorporation will then reflect an innovative and sustainable approach that fosters resource efficiency, reduces waste, promotes eco-friendly construction practices, and contributes to the circular economy by transforming waste into a valuable resource. Fig. 1 shows various ways how FDS can be applied in building materials [30,88]. Specifically, FDS can be used as a natural aggregate replacement or an alternative to clays used for the production of bricks or compressed earth bricks. If calcined at high temperatures, FDS can be applied as a supplementary cementitious material for partial cement replacement or even as a complete cement replacement for the design of alkali-activated materials.

Several influential factors guide the use of FDS in building materials, encompassing environmental, economic, and technical factors. Material properties, such as physical and chemical characteristics, and water content, significantly influence its suitability in this case. Low water content and uniformity in FDS composition are vital for maintaining consistency and performance, and environmental regulations governing contaminants levels are crucial for environmental protection [88,89].

The economic feasibility of FDS-based building materials when compared to traditional alternatives plays a pivotal role in their adoption within building projects. Implementing methods to reduce production costs such as an efficient dewatering process can enhance its economic feasibility, while the environmental impact and its contribution to sustainable practices influence its overall acceptance. Locally available FDS may reduce material transportation, thereby reducing carbon footprint. Importantly, structural integrity and durability are crucial for successful integration [52,90]. Addressing these factors collectively underscores the successful valorization of building materials, which can promote sustainability and innovative practices within the construction industry [88]. The amount of Al_2_O_3_ can be deemed beneficial thanks to the vitrification processes during firing and the transformation into mullite with high mechanical strength. In terms of securing the desired material parameters, kaolinite and illite represent important mineral phases that contribute to the preservation of mechanical properties after exposure to increased temperatures as those minerals are similar to the original clay used for the brick production [52,91].

The objective of this study is to uncover the diverse advantages of using sediments as a sustainable building material by thorough examinations of sediment applicability, complemented with in-depth descriptions. To unlock the full potential of sediments in that respect, it is necessary to understand how sediments behave when incorporated. Therefore, the insufficiently comprehensive and very fragmented information describing the possibilities of recycling FDS in the construction industry will be analyzed first and foremost in this review. Its main purpose is to outline the potential use of FDS in the construction industry, including the achieved results, and use them to determine key areas and challenges.

### 3.2 Fired bricks

The primary materials used in brick production are clay minerals mixed with additives for improved properties like plasticity and workability [92]. Approximately 4 billion tons of clay resources are extracted annually for clay brick production, the extensive exploitation of clay resources has given rise to ecological challenges, including vegetation destruction, erosion, pollution, and land degradation which has prompted certain countries to impose restrictions on clay brick usage to conserve resources and protect the environment [43,93,94].

Similarly, the brick industry is known for its substantial energy consumption, leading to the release of significant pollutants into the environment [95]. For instance, fired clay bricks exhibit the highest energy consumption among similar products in the industry, with 96 % of this energy consumption occurring during the manufacturing process [96]. The carbon emissions associated with their production have raised concerns about their environmental safety, prompting new research into eco-friendly approaches for producing unfired bricks. In this regard, several types of waste materials have been investigated as alternative materials for brick production to reduce natural clay resource dependency. Such waste types include paper production residues [97] spent foundry sand [98,99], saw dust [[100], [101], [102]], fly ash [103], limestone powder [102], steel slags [104,105], or agrowaste [106,107]. Few studies have been conducted on the incorporation of FDS [85,[108], [109], [110]].

### 3.3 Unfired bricks

In the quest to design novel bricks, opting for FDS can mitigate disposal challenges and reduce raw material costs. Unfired bricks from FDS are usually produced by mixing raw FDS with reinforcement such as soil waste, minerals, or agrowastes, compacted and sun dried to produce durable earth bricks. Research introduced by Maierdan et al. [95] described the utilization of raw FDS, where natural fiber (oil palm flower) was used as reinforcement in the manufacturing process for lateral loaded walls, emphasizing the economic viability of FDS in producing unfired bricks. The unconfined compressive strength (UCS) of the brick specimens was 2.8 MPa. Bhairappanavar et al. [96] noted that the earth brick made from FDS and compacted with hemp shiv reached a satisfactory flexural strength in terms of recommended strength limits. Therefore, FDS in unfired brick production emerges can be considered as a promising and sustainable option for the building industry as it is possible to reuse raw FDS with reduced energy consumption.

### 3.4 Compressed unfired blocks

Blocks as versatile building materials play an important role in load-bearing and partition walls, as well as in constructing exterior walls for building envelopes. They also aid in sound insulation between rooms or from external sources. Building blocks are made from diverse materials to achieve different sizes and shapes that offer design and construction flexibility. They are of different types including hollow blocks, solid, cellular, sand and cement blocks, compressed earth blocks (CEBs), etc. [111,112].

Compressed earth blocks (CEBs) have gained potential as an alternative building material. They are commonly produced by combining local soil, stabilizers, and water under pressure to create high-density blocks. The strength and durability of CEBs depend on soil composition, plasticity, and clay content. Optimal clay content falls within the 5–20 % range, with lateritic soils proving most suitable for enhanced strength and durability [[113], [114], [115], [116]].

CEBs offer numerous advantages, as they can be locally produced using simple construction methods without the need for specialized equipment, providing excellent thermal and acoustic insulation. They are approx. 2.5 times larger than conventional fired clay bricks, which speeds up construction processes. Additionally, CEBs do not require firing for final strength, resulting in lower energy consumption which makes them cost-effective and affordable [112,114,115,117,118].

Despite the cost-effectiveness of earthen construction, CEBs face challenges, such as a lack of standardization in many countries, limiting their widespread adoption. Variability in soil characteristics affects CEB mix designs, impacting their ultimate behavior. Furthermore, CEBs exhibit limitations, such as low strength, susceptibility to water, poor impact and abrasion resistance, low fire resistance, high porosity, and low durability [112,119,120]. Cement stabilization has been accepted as a common solution for material improvement. However, the environmental impact of cement production, including CO_2_ emissions, remains a challenge [121,122]. Alternative materials are therefore considered; clay is an essential natural binder but a high content of clay in CEB can lead to shrinkage and cracks [123]. For the sake of sustainability, incorporating waste materials, minerals, or agrowaste is suggested to reduce energy demand and contribute to the circular economy of CEB production. Various researchers have explored the use of silica-alumina-rich waste materials, such as fly ash, sawdust ash, zeolite, rice husk ash, blast furnace slag, and FDS, as a replacement for cement in CEB [30,124,125]. The incorporation of these materials has demonstrated social, economic, and environmental benefits, by maintaining low production costs and minimal environmental impact [120,126,127].

FDS with sufficient content of SiO_2_, Al_2_O_3_, and CaO in amorphous form may provide pozzolanic activity to use the material as a partial cement replacement in CEB production. Here, usually amorphous portion higher than 20 % allows a minor cement replacement in case of advanced mixture design [128]. However, the inherent properties of FDS, including natural constituents and possible contaminants like toxic elements and organic matter can negatively affect strength development [30]. Considering these factors, it is essential to evaluate the feasibility of converting FDS into FDS-based CEB for various applications. This involves validating the leachability of contaminants from the blocks against required environmental standards and assessing the economic applicability of FDS-based CEB by comparing their market competitiveness before implementing them on a field scale [129].

The prospective use of FDS for block production holds significant promise in fostering sustainable and eco-friendly construction practices. This alternative approach effectively addresses the depletion of natural resources by repurposing abundant FDS, thereby reducing reliance on conventional materials, and converting potential environmental hazards into valuable resources in line with circular economy principles [112,114,123]. The widespread availability of FDS has the potential to contribute to cost savings in block production. As manufacturing processes evolve in the future, incorporating FDS processing techniques could further enhance the quality and performance of produced blocks.

### 3.5 Aggregate

Aggregate is an important component used for concrete production. According to the origin, aggregate can be sourced either from nature or manufactured. Natural aggregate consists of materials, such as sand and gravel, commonly sourced from natural locations like riverbeds, floodplains, and coastal areas. Additionally, crushed stones like granite, limestone, and basalt are extracted from rocks by quarrying [130]. Over time, there has been a growing demand for these materials by construction industries, resulting in the depletion of raw materials, and vegetation and harm to ecosystems**.** Natural aggregate materials are getting scarce and expensive, prompting an increase in government pressure to seek alternative solutions by identifying new sources [131,132].

#### 3.5.1 Natural aggregate

Considering the FDS potential for the aggregate production, researchers have explored sieve and wash techniques to obtain coarse aggregates, sands, and fines from FDS, providing essential elements to formulate various concrete mixes. Such utilization contributes to the efficient use of available natural resources and addresses environmental concerns [3,133].

Several advantages are derived from the use of FDS as a replacement for natural aggregate apart from natural resource conservation, it promotes the circular economy principle, saves cost, and its adaptability when processed properly making it a versatile option for construction projects with specific requirements. Similarly, it addresses the disposal issue by reducing the environmental impact associated with the disposal in landfills or aquatic environments. Also, FDS for building purposes might not require any special treatments depending on the level and type of contaminant present in it [27,133,134].

The potential of FDS as aggregate material was studied by various researchers. Couvidat et al. [135] utilized sieved FDS with a particle size of 80 μm as the fine aggregate in mortar. They observed a significant improvement in the compressive strength of concrete. Beddaa et al. [3] investigated the feasibility of producing C30-class concrete using FDS as aggregate. Their findings indicated that a slight increase in cement dosage (less than 5 %) resulted in comparable properties to the control sample**.**

#### 3.5.2 Artificial aggregate

For the sake of sustainability, the replacement of natural aggregates with non-conventional alternatives, such as manufactured aggregates [136,137], and manufactured sand [138,139], are being explored. Aggregates can be manufactured from raw FDS via the pelletization process. FDS is mixed with cementitious material as a binder and pelletized to form a dense construction material. Performance evaluation of using raw FDS in manufactured aggregate where the correlation between the microstructure characteristics and mechanical properties of FDS used in manufactured sand concrete (MSC) was investigated by Huang et al. [140]. When the content of FDS was 25 %, MSC exhibited better mechanical properties and work performance. It was reported that adding a desired amount of FDS can increase the density of MSC and reduce porosity, improve the uniformity of pore distribution and the structure of the interfacial transition zone, and optimize the phase composition. Wan et al. [141] reported a cylinder compressive strength of about 6.5 MPa using FDS aggregate.

Despite the apparent potential, there are significant gaps in the utilization of FDS as aggregate. The engineering properties of FDS may not align with those of traditional aggregates. For example, high water absorption can hinder effective bonding between binder and aggregate phases, ultimately compromising the strength and durability of concrete. The variable composition of FDS from different sources causes challenges in ensuring consistent quality. Low quality of aggregates results in high porosity in concrete, raising concerns about durability. Achieving the desired particle size distribution may necessitate additional processing, impacting overall feasibility and cost. Furthermore, FDS may contain contaminants like heavy metals or pollutants, imposing limitations on its use as building material. This requires careful treatment, particularly when incorporating sand from marine FDS into concrete, as the high chloride ion content can induce erosion in buildings, leading to decreased performance [140,142]. Additionally, a low understanding of the long-term performance and behavior of FDS-based aggregates serves as a barrier to widespread commercialization. Project managers, regulators, and contractors may perceive long-term risks associated with the use of FDS-base aggregate as building material [89,143]. Discrepancies in national regulations could impede international markets due to legal inconsistencies between countries. Thus, variations in specifications pose resistance to a broad adoption of FDS as an alternative aggregate material [89].

Nevertheless, the growing emphasis on sustainability in construction might promote FDS as an important material in eco-friendly building construction practices. Enhanced processing techniques could improve the quality and uniformity of the aggregate, making it a more viable alternative to natural aggregates. While the establishment of regulations and standards for FDS-based aggregates in building materials could promote widespread adoption, strict regulations might hinder progress by restricting economic, technical, and environmental feasibility [48,89]. Therefore, for FDS-based aggregates to become an essential material in building construction, careful consideration is required.

#### 3.5.3 Lightweight aggregate

Lightweight aggregates (LWA) are granulated materials with a density below 2000 kg/m^3^. The versatility of lightweight aggregates finds use in a diverse range of constructions. They are used in the production of lightweight concrete with the benefits of improving the structure durability, reducing structural loads, and improving thermal insulation of buildings [48,144,145]. Lightweight aggregates also find use in geotechnical applications, drainage systems, and as components of lightweight structural elements for both residential and commercial buildings. The reduction in the total weight of concrete when incorporated with LWA, is useful for applications, such as lightweight floors and walls, facades, and signs [144,146].

Since FDS consists of minerals, such as aluminosilicate, quartz, and feldspar similar to clay, it can be used for LWA production. This facilitates the repurposing of FDS as a substitute for clay in LWA production, reducing the reliance on scarce clay resources [87,141,147]. This not only enhances the sustainability of LWA but also adds value by diverting FDS from landfilling. Two technical approaches have been developed to produce LWA from FDS, namely sintering (heating) and non-sintering (cold binding) methods [87,[148], [149], [150], [151], [152], [153]].

Sintering technology has been the most renowned method for LWA production, however, the method is not eco-friendly and unsustainable due to high energy costs and air pollution associated with this technology which cannot be overlooked [154].

Cold bonding stands as another non-sintering technique for LWA production. It allows the formation of LWA at room temperature and thus provides economic and environmental benefits compared to the sintering technology [95,152,[155], [156], [157]]. In cold bonding technology, cement, or alternative materials can be utilized as binders within the pelletization process [157]. In the relation to environmental concerns associated with cement production, supplementary cementitious materials are widely adopted to reduce carbon dioxide emissions and improve the material durability. Research performed by Peng et al. [158] recommended the optimum material composition of FDS (80 wt%), cement (3 wt%), quicklime (3 wt%), gypsum (3 wt%), fly ash (5 wt%), and water glass (3 wt%) in their study to achieve the compressive strength of about 2.46 MPa. Reduced cement consumption is a first step toward sustainable LWA production by using an economical and environmentally-friendly cold bonding method. However, further research is essential to identify the most efficient approach for producing LWA from FDS using this method. Despite the challenges and the limitations, the utilization of FDS for LWA production offers numerous benefits. With continued research, technological advancements, and regulatory support it holds significant future potential to produce sustainable and cost-effective building materials.

### 3.6 Blended cements

The replacement of Portland cement in concrete poses one of the major issues in current environmental engineering challenges related to the building industry. The global Portland cement emission of CO_2_ accounts approx. 8 % of global emissions, thus the efficient reduction scenario is of particular importance. Based on the available knowledge, the utilization of the calcined clays is one of the possible scenarios applicable for lowering the clinker ratio in cement. In this regard, the FDS with content of clayey minerals can be considered as an abundant alternative to clays, which can concurrently solve the problem associated with the FDS disposal. FDS, depending on the location and containment of the sandy phase, may contain a significant portion of kaolinite, smectite, and illite. Kaolinite, a vital component of clays, exhibits high pozzolanic reactivity when subjected to calcination [4,88]. Utilizing FDS in cement production can be a cost-effective alternative source of raw material as FDS are abundant and readily available waste material. Replacing a portion of clinker with FDS will reduce the demand for limestone, contributing to sustainable resource management with a positive environmental impact. High energy demand and carbon footprint associated with cement production will also be reduced [159,160].

The hydration reaction in blended cements replaced with FDS leads to the formation of calcium silicate hydrate (CSH) and calcium aluminate hydrate (CAH), so the formed hydration products contribute to the strength, durability, and resistance of the binder making it suitable for various applications [21,161]. Dalton et al. [162] discussed the potential of FDS for semi-industrial scale replacement of Portland clinker according to the presence of SiO_2_. Here, the replacement up to 39 % was found feasible. The mechanical strength corresponded well to the reference sample composed from cement only at an early age (3 and 7 days), and even strength increase by 20 % was attained at later ages (28 days). On the other hand, Faure et al. [82] suggested using only 10 %–15 % as a Portland clinker substitute for the preservation of concrete performance. In comparison to limestone filler, the effect on hydration was even improved by using uncontaminated FDS, as reported by Zhao et al. [58].

Considering the environmental point of view, the utilization of FDS as an alternative constituent of blended cement may provide a reduction in CO_2_ emission. Moreover, the waste disposal reduction and limited dependency on natural raw materials may further improve the ecological footprint. Apparently, the utilization of FDS promotes the principles of circular economy.

However, the potential presence of harmful heavy metals like leachable lead and cadmium in this material raises environmental concerns as it meets criteria given in legislative (Regulation (EC) No 1272/2008 of the European Parliament and of the Council of December 16, 2008 on classification, labeling and packaging of substances and mixtures). Their impact on the environment must be carefully considered when FDS is utilized [155]. In addition, meeting quality standards for commercial products remains a challenge as the use of FDS often results in inferior physical properties of composites compared to more conventional alternatives [134,147,152].

### 3.7 Alkali activated materials

**Fig. 2:**
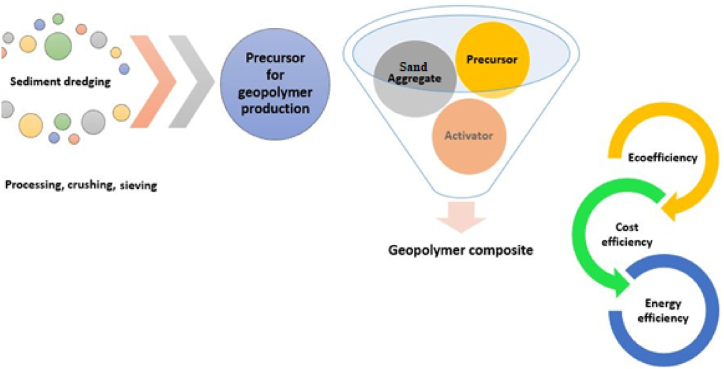
Simplified scheme of alkaline activation of FDS.

To mitigate cement-related emission problems, cement-free binders were developed using alkaline activation of suitable materials [163]. Similarly to common precursors, such as slag or fly ash, FDS can undergo a reaction with alkaline solutions (sodium silicate, sodium hydroxide) resulting in the formation of geopolymer matrix and providing more pronounced savings in CO_2_ emissions compared to partial cement replacement (see scheme in Fig. 2). However, the readiness of this technology is far from the construction practice even for the more intensively studied precursors. The research aimed at this field thus represents an open challenge [79].

Only a few studies focused on the alkaline activation of FDS have been published to date. Many of these examine the valorization of calcined FDS at a temperature range of 400 °C–900 °C [77,79,164]. It was found that an increase in calcination temperature increases the reactivity of raw FDS, however, excessive energy required to attain the calcination goal is not cost-effective with detrimental CO_2_ emission. In this sense, efficient reuse of raw FDS is currently investigated by direct co-valorization with other byproducts. For example, using FDS in combination with geopolymer material enhances the mechanical properties of building blocks, providing resistance against environmental factors like rain, frost, wind, or temperature changes [113]. Ferone et al. [164] successfully produced AAMs with a low carbon footprint by incorporating 30 % of high organic matter FDS into GGBS activated with a commercial alkali activator. The authors reported UCS above 2 MPa. However, further investigation with the addition of 3 % of calcium carbonate improved the UCS by 90 %. As recognized, it is possible to replace GGBS up to 70 % with FDS of low organic matter. Monteiro et al. [51] evaluated the impact of mortar developed from untreated FDS having 30 % water content, co-valorized with GGBFS and activated with 4M NaOH/Na_2_SiO_3_ solution and cured at ambient temperature for 28 days, and compared the results with cement stabilized FDS. A compressive strength of 3.95 MPa was observed and the reaction effectively reduced the impact on climate change by 66 %, taking FDS beneficial recovery into account.

A further improvement was achieved by Fořt et al. [79], who used potassium water glass as an activator and reported a compressive strength of almost 37 MPa. Here, kaolin and illite appeared as indicators of suitability for the alkaline activation. The development of mechanical strength is also correlated with the Si/Al ratio and a sufficient availability of Na^+^ and K^+^ ions. The low silicate modulus of the applied activator and high sodium silicate content were found as the main reason for poor mechanical strength by Zhou et al. [165]. On the other hand, a proper mix design could deliver applicable strength levels, and provide considerable CO_2_ emission savings (20 %–36 %) compared to cement alternatives. Similar conclusions were formulated by Alloul et al. [166], who also noticed the possible immobilization of heavy metals by alkaline activation of contaminated FDS.

The benefits associated with the immobilization of hazardous compounds studied by Tian and Sasaki [167] uncovered the potential of geopolymers in the field of environmental utilization of FDS. Specifically, Cs^+^ and SeO_4_^2−^ were sorbed into the structure of geopolymer and this application could be further extended for the stabilization of radionuclides. As concluded by Ji and Pei [168], despite various issues related to the stabilization of heavy metals by geopolymers, this approach possesses promising results, and such oriented research may provide additional application value.

In a nutshell, while there are notable benefits in using FDS as a binder component, the application is not without limitations and challenges. FDS can exhibit variability in mineral composition, contaminant levels, and quality, which may affect the binder properties [169]. Achieving a specific particle size distribution is crucial for optimal binder performance; obtaining this may require additional processing steps. Meeting quality standards and regulatory requirements for commercial binders can also be challenging due to potential variations in FDS properties. FDS-based binders may exhibit poor properties, such as durability and strength compared with conventional binders. In addition, challenges such as public perception need to be addressed for the full realization of its potential [48,170,171]. Further research and commitment to sustainable practices will play a crucial role in shaping the future of binder production using FDS.

## 4 Benefits, limitations and future outlook

The advancement in FDS treatment technology for disposal or reuse purposes can contribute to more sustainable and environmentally friendly disposal practices compared with traditional methods, such as direct open dumping, river, and ocean disposal, landfill, land reclamation without remediation, or direct agricultural applications [[172], [173], [174], [175]]. Owing to the mineral composition FDS can be processed as binder components, which is the best upcycling option, but they need to undergo a combination of processing techniques to address potential issues that could otherwise compromise the quality [21,31,176]. While FDS-based materials hold promise in building construction, it is essential to explore formulations that strike a balance between the properties of FDS and other components. Researchers and engineers can capitalize on the diversity in FDS composition to tailor them to meet specific attributes, such as strength, durability, or thermal insulation capability. This approach optimizes the utilization of available resources, while promoting circular economy principles [131,177].

**Fig. 3:**
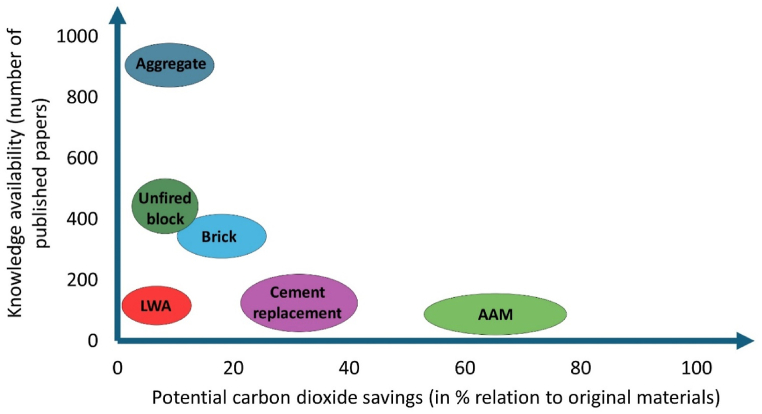
Comparison of selected utilization scenarios of FDS in environmental and knowledge availability scale.

The precise assessment of environmental profiles of alternative materials based on FDS is relatively rare. The general relation between the occurrence of the research topic in the Web of Science papers, and the potential environmental benefit expressed by CO_2_ savings are included in Fig. 3. In general, the replacement of low-embodied energy materials, such as natural aggregate, does not provide significant environmental advantages for natural resources. A major benefit related to the replacement of such materials lies in the limited availability of raw materials in various regions as well as the synergistic solution between the WS disposal requirements and the valorization as secondary-raw material. Accompanied by the large quantities produced within the dredging operations, it may be viewed as a valuable and abundant source of material. On the other hand, long transportation distances significantly hinder the environmental benefits similar to other natural aggregate alternatives, as concluded by several authors [178]. The local availability of the material may provide additional benefits and lower the environmental load related to the disposal of FDS. In addition, the valorization of such waste/by-product material contributes to the circular economy upcycling principles and preserves the natural materials [179]. The application of treatment processes further worsens the environmental profile of such material. For example, the research performed by Svensson et al. [180] on marine sediment disposal options shows significant effects of electrolysis treatment of polluted sediments to extract the metals. However, this study does not include the avoided production of raw materials into consideration, and the comparison with other authors is complicated. The utilization of WS as a source for the production of lightweight aggregates poses more advanced applications. However, the temperature required for expanding the material can be deemed as the environmental and cost barrier, as the acceptance of alternative aggregates by end-users is limited.

The avoided processes play an important role in the carbon footprint calculation (or LCA). The replacement of more energy-intensive materials provides more distinct benefits and significant carbon dioxide savings. As reported in Bozic et al. [62], the replacement of the clay for the production of the fired bricks up to 30 wt% resulted in 12 % savings excluding the prolonged transport distances. The content of the CaCO_3_ negatively affects the CO_2_ emissions as a consequence of the limestone decomposition. As noted, the identification of a closer source of the WS is of particular importance to achieve notable savings [62].

Principles of circular economy and upcycling prioritize the replacement of energy-intensive materials. As the production of cement is responsible for about 8 % of CO_2_ emissions, the reduction of its use represents a major research task. Therefore, the processing and transformation of WS into a binder provides more evident environmental benefits. Such valorization requires a high-temperature treatment (between 650 and 900 °C) which worsens the environmental footprint. However, the benefits that arise from the avoided production of traditional materials may fully compensate for this issue. The calcination of WS utilizes the content of clay minerals (kaolinite in particular) and converts it into metakaolin which can react with calcium hydroxide in blended cements. Such a solution provides significant savings due to the replacement of Portland cement up to 40 % (depending on the strength grade), which accounts for 95 % CO_2_ emissions of concrete. Moreover, metakaolin can be applied as a precursor for the alkaline activation and formation of geopolymer structure with the capability of 100 % replacement of cement.

As described in Ref. [181], the barriers from the customer perspective are related to technical and environmental impact. However, the price of the materials can substantially affect the decision process and the acceptance of such materials if the environmental declaration is provided. The investigated example was carried out on the issue of bricks. However, the situation with concrete or AAMs with sediment content is more complicated due to the immobilization capability of these materials. In particular, project owners and design teams struggle to clearly define the parameters needed to assess material safety. The financial burden of these required studies can be substantial and without guaranteed validation from regulatory agencies this uncertainty may discourage further project development. As a result, the effective utilization of dredged sediments faces significant challenges stemming from various uncertainties. A summary of the most critical areas in terms of benefits, disadvantages, challenges, and future potential is provided in Table 3.

**Table 3:** Summary of most crucial factors related to FDS reuse.

Benefits	Disadvantages
1 Using sediments in constructions enhances resource efficiency and provides environmental benefits. 2 Sediments are accessible locally; they present a low-cost option by minimizing the requirement for transportation. 3 Employing sediments responsibly aligns with circular economy principles by rationalization of material flows and reduction of waste generation. 4 Immobilization of heavy metals in alkaline environment of FDS-based composites further improves the environmental issues. 5 Upcycling the FDS through additional processing provides synergy between preserving the water reservoirs and dams function and concurrently reducing dependence on natural raw materials.	The quality of sediments can exhibit significant variations, creating difficulties in maintaining uniform characteristics for construction purposes. Sediments may contain contaminants like heavy metals, pollutants, or pathogens that can cause environmental and health hazards, thereby public resistance may arise due to apprehensions about environmental and health consequences. The utilization of sediments in construction is subject to stringent regulations and requires permits which can be time-consuming to process. Challenges related to stability, compaction, and adherence to standards can be posed by sediments for some construction applications.

**Future potential**	**Challenges**

1 Continuous improvement in sediments' management practices can increase their potential use in constructions. 2 Increasing awareness of sustainable construction practices can enhance the future acceptance of sediments in construction materials. 3 Utilizing sediments in the construction industry will contribute to sustainable and environmentally friendly construction practices.	1 Testing and analysis are essential to evaluate the quality of sediments, which can demand significant resources. 2 The transportation of sediments to construction sites can be challenging, particularly when suitable sediment locations are not in proximity. 3 Overseeing the environmental impact of sediments' use, which includes addressing runoff and mitigating ecological disturbances, can present another challenge. 4 Achievement of satisfactory mechanical properties requires further focused research to provide competitive materials.

## CRediT authorship contribution statement

**Jan Fořt:** Writing – original draft, Supervision, Project administration, Methodology, Investigation, Funding acquisition, Formal analysis, Data curation, Conceptualization. **Ayodele Afolayan:** Writing – original draft, Investigation, Formal analysis, Data curation. **Václav Kočí:** Visualization, Validation, Software. **Lenka Scheinherrová:** Project administration, Methodology, Investigation. **Jiří Jan:** Validation, Resources, Investigation, Conceptualization. **Jakub Borovec:** Writing – original draft, Supervision, Project administration, Methodology, Investigation. **Robert Černý:** Writing – original draft, Supervision, Project administration, Formal analysis.

## Data and code availability statement

Data included in the article material is referenced in the article.

## Declaration of competing interest

The authors declare that they have no known competing financial interests or personal relationships that could have appeared to influence the work reported in this paper.
